# Ensuring Corporate Security and Its Strategic Communication in Healthcare Institutions in Slovenia

**DOI:** 10.3390/healthcare11111578

**Published:** 2023-05-28

**Authors:** Valentina Kubale, Teja Lobnikar, Branko Gabrovec, Miha Dvojmoč

**Affiliations:** 1 Veterinary Faculty, University of Ljubljana, Gerbičeva 60, 1000 Ljubljana, Slovenia; 2 Faculty of Criminal Justice and Security, University of Maribor, Kotnikova 8, 1000 Ljubljana, Slovenia; 3 National Institute of Public Health, Trubarjeva 2, 1000 Ljubljana, Slovenia

**Keywords:** corporate security, healthcare, strategic communication, Slovenia

## Abstract

Ensuring corporate security is an essential and critical component of any healthcare facility to provide safe services to its patients and employees. Healthcare facilities must employ a variety of strategies to ensure corporate security. This includes developing a comprehensive communication plan that defines the roles and responsibilities of the various stakeholders. The objective of our study was to present the concept of corporate security in healthcare institutions and in the Slovenian healthcare system, to highlight current threats in healthcare institutions in Slovenia, to describe the importance of strategic communication of corporate security in healthcare, and finally to define the current state of corporate security in Slovenian healthcare institutions in Slovenia. A survey was conducted and distributed to healthcare institutions in Slovenia to obtain results. A total of 154 healthcare stakeholders participated in our study. The results showed that corporate security is present in Slovenian healthcare facilities, but additional efforts are needed to improve it, especially considering the current challenges related to the measures taken after the COVID-19 epidemic and the shortage of healthcare personnel. The legal processes of corporate security in healthcare facilities comply with applicable laws and regulations to protect the interests of their patients and employees. Operational security processes are currently provided primarily by internal providers. There is a need for improvement, particularly in the training and education of staff, who play the most important role in ensuring safety. To effectively establish comprehensive corporate security, strategic communication with all stakeholders is essential to ensure that their security policies and procedures are properly implemented.

## 1. Introduction

We live in a time when security has become an important value that governments, as well as individuals and institutions, do not take for granted. Security is one of the most important foundations for the unhindered functioning of the state and is especially important for ensuring a safe environment for the development of society [1]. The current health crisis has shown how vulnerable this value can be and how important it is to ensure it, especially in institutions that are responsible for the social security of all of us. The security of healthcare institutions and the potential collapse of the healthcare system are currently important public issues, as the crisis has shown the importance of organizing and ensuring stability and security [2].

As healthcare facilities increasingly face risks to the safety of employees, visitors, and patients, as well as to the safety of operational procedures, establishing comprehensive corporate security is important to adequately ensure safety at all levels. Ensuring adequate corporate security in healthcare is a complex process that requires extensive knowledge and processes that are implemented as a function of all other key corporate functions. The mission of corporate security is to protect the organization, its technology, employees, patients, technical resources, and customer data from internal and external threats [1,3]. Organizing corporate security in healthcare institutions requires specialized knowledge reflected in six main processes that must be performed at a high level, comprehensively, and based on expertise. These processes include (i) the legal assurance of lawful and unhindered operation, (ii) legal assurance of organization-specific security know-how, (iii) legal and physical protection of technologies and the information system (IT security), (iv) legal protection of material and intellectual property rights, (v) assurance of personal protection measures in terms of state regulations, and (vi) provision of occupational health and safety activities [4].

Corporate security, implemented in line with and in parallel with other operational processes of healthcare institutions, is also important to achieve the organizational goals without suffering harm, whether financial, material, or within the operational processes themselves [1]. In today’s world, organizations are exposed to numerous dangers and threats in their operations, especially cybercrime, physical and verbal aggression, and errors resulting from human negligence. Corporate security provides protection against such risks [5]. However, for corporate security to be effectively implemented, it must be strategically communicated. All stakeholders in healthcare need to be clearly informed about how each legal and business process works [6], and their benefits and importance to security must be demonstrated through communication, which can help reduce risk [7]. In organizations (including healthcare), the weakest link in the security chain is usually the human [8].

The aim of this article is to present: (i) the concept of corporate security in healthcare institutions, (ii) the concept of corporate security in healthcare institutions in the Slovenian healthcare system, (iii) highlight current threats in healthcare institutions in Slovenia, (iv) to describe the importance of strategic communication of corporate security in the healthcare system, and (v) define the current of corporate security in Slovenian healthcare institutions in Slovenia. The article focuses mainly on the mechanisms for ensuring corporate security, the state of corporate security in the healthcare system in the past and today, and the challenges and proposals for the future. The article presents the results of a survey conducted in Slovenian healthcare institutions in 2022, with the participation of 154 healthcare stakeholders at primary, secondary, and tertiary levels.

### 1.1. Corporate Security in Healthcare Institutions 

There are different definitions of security, and in its most basic and objective sense, we can understand it as the absence of threats to acquired values, while in the subjective sense, security is an absence of fear in the face of threats [9]. 

Corporate security is an activity that identifies and implements all necessary systemic measures to ensure security in organizations, protection of property and also business operations, and control security threats in an individual company [10]. Corporate security is integrated and involves the implementation of several different functions that must be aligned [3]. These include the area of security, the process of continuous operations, security IT, protection of the company’s vital information, security of employees, and workplace security. Elements of the security risk assessment model include risk identification, analysis, assessment and control, risk reporting, and risk assurance. Thus, the definition and management of security risks require the development of a specific plan, which must also include legislation and a code of ethics in accordance with existing practice [5,10]. The processes of corporate security are a totality of the corporate model of corporate governance, which manifests itself primarily in corporate governance and corporate social responsibility [5,10]. It is important for healthcare institutions to be aware of the real risks and therefore establish a good system of corporate security, as this will make their work more successful and effective, and most importantly, safer for all [10]. Security is a good that gives us a sense of safety, and corporate security is a set of security measures developed to ensure security in a particular environment [11,12,13].

In its broadest sense, corporate security is an activity of identifying and implementing all necessary systemic measures to address security risks in an individual organization. It is a set of activities that contribute to the security of the company and the prevention of internal and external threats, based on well-educated, trained, and experienced personnel, of course, in cooperation with all other key functions in the company [4,5]. The purpose of corporate security is to ensure order, compliance with laws and internal regulations, and the safety of people and property in the organization [10,11]. 

In defining security, it is important to mention the need for safety and security, which was recognized as a basic human need by Abraham Maslow in his ’Hierarchy of Needs’. This motivational theory states that human needs can be placed in a hierarchy in which basic physiological needs such as food, water, and shelter are at the bottom, followed by safety needs, social needs, esteem needs, and self-actualization needs at the top. The security and safety needs, which include physical safety, stability, and security, are at the second level of Maslow’s hierarchy, just above the physiological needs, and include job security, financial stability, and protection from physical harm. According to Maslow, safety and security needs must be satisfied before a person can progress to higher levels of need. Without a sense of safety and security, a person may feel anxious and unable to focus on higher needs [14].

Healthcare institutions are an important component of a country’s social security, as their work ensures the safety of all citizens [13], and we are far too rarely aware of the importance of their stability and integrity. Healthcare institutions must always function smoothly, regardless of unforeseen situations [15], and all employees, as well as patients, must feel safe in them. For anyone entering a hospital or healthcare institution to receive a healthcare service at any level, concern for safety must be the least of their worries. Healthcare workers deserve the highest level of protection as they serve their communities and the people entrusted to their care. Patients are in vulnerable positions in facilities and should receive an appropriate level of security from their caregivers and healthcare institutions. For these reasons, most hospitals take security very seriously [7]. In addition, hospital security policies are becoming more stringent to address new security threats [8]. As security threats become more common, organizations are becoming increasingly aware of the importance of corporate security. 

### 1.2. Ensuring Corporate Security in Healthcare Institutions and the Situation in Slovenia 

Corporate security through corporate security management is an integral security system, which is also of great importance in Slovenia and provides one of the security, economic, and business benefits in a healthcare institution [1,5,16].

To better understand the different levels of the Slovenian health care system, we would like to briefly explain the system regulation. The healthcare system in Slovenia is divided into primary, secondary, and tertiary levels. The primary level of the Slovenian healthcare system includes primary healthcare and pharmacies. It is important that primary healthcare is accessible in the shortest possible time, taking into account the geographical distribution, the distribution of primary healthcare, and the appropriate time periods for accessing healthcare services [17]. The secondary level is an extension of the primary level of healthcare and includes the operation of specialized outpatient clinics and specialized hospitals, while the tertiary level includes the activities of clinics, clinical institutes, or clinical departments. Emergency medical care is also part of primary and secondary healthcare. Emergency medical care includes individuals who are appropriately qualified to provide first aid at the location of an injury or serious event that requires the use of emergency medical services [17]. At the tertiary level, the University Medical Centre Ljubljana and the University Medical Centre Maribor operate, conduct research and perform teaching activities for the Faculty of Medicine and other university and technical colleges and provide the most sophisticated outpatient and inpatient healthcare services [17]. Public health activities also include the public health sector, including the National Institute of Public Health, as well as all public health-related activities in the fields of health, environment, and nutrition [17]. The sum of the levels described above constitutes the entirety of the public health system, although there are also some private health institutions in Slovenia.

Security, especially personal protection, in the Slovenian healthcare system, is provided mainly by private security companies operating on the basis of the Private Security Act [18]. These companies are responsible for protecting the property of healthcare institutions and ensuring the safety of all employees, visitors, and patients at all levels of the healthcare system. In the event of external or internal threats, security is reinforced not only by private security companies but also by detectives and, in the case of security planning, by the Civil Defence Organization. In healthcare, employee safety is extremely important and is ensured through a safe workplace as part of corporate security, which includes legal requirements for individuals and personal data, the work environment, and workplace health and safety design [4].

Corporate security also includes the detection of fraud and other criminal activities. When a security company detects fraud or other form of crime, it must immediately notify the police, who will then prosecute the case. As part of corporate security, organizations must develop preventive measures to eliminate all risks, reduce threats to the lowest possible level, develop an operational plan for crises, increase competitiveness and productivity, improve technology, and act when security threats occur [19].

To ensure effective and adequate corporate security, organizations must establish a corporate security management system [11]. This is used to ensure an effective mechanism of risk management. Due to their complexity and the importance of ensuring continuous operation, healthcare institutions have a dedicated corporate security department headed by a corporate security manager who can address the internal and external risks in a given organization. It is important that the person performing this role has a good understanding of security threats while having the necessary expertise to manage this complex activity. The corporate security manager aims to improve the performance and efficiency of the organization’s operations, prevent safety risks, and improve the safety culture of all employees [20]. Very important is the professional training of the security manager, whose duties include the preparation of the security plan, which is the basis for the corporate security policy in the organization. The security plan contains a snapshot of the existing situation, an analysis of the existing situation, and suggestions for security improvements. Based on the security plan, further security measures in the organization are determined. In order for the organization to manage successfully and efficiently, it is necessary that the person responsible for security is committed to the organization and does not act maliciously [1]. Through their strategy and technical means, corporate security managers must clearly identify and, above all, prevent security threats [4]. 

To achieve effective corporate security, an organization, such as a healthcare institution, must successfully implement physical security, technical security, an early warning system, an effective risk management system, and specific procedures for conducting security checks and controls [10]. Furthermore, the security management of a company must include security knowledge as well as knowledge of law, entrepreneurship, IT, finance, psychology, and organization. The company’s security experts must also conduct internal audits of security risks and compliance with ISO standards so that the company can maintain its security function [21]. 

Corporate security also plays an important role in improving the security culture among employees, which is crucial because the employees of healthcare institutions can be the strongest link, but they can also be the easiest target of risks if there is insufficient information and awareness [10,20].

### 1.3. Current Security Threats in Healthcare Institutions in Slovenia

Countries, institutions, and individuals are threatened by a variety of situations and circumstances [22]. We are particularly threatened by unforeseen situations beyond our personal control, such as the financial crisis, violence, and the health crisis [9]. The pandemic, which was originally a health problem, led to a widespread health crisis, and healthcare institutions became a key factor in addressing this situation, which also had an impact on the security of healthcare institutions. 

Based on the Critical Infrastructure Act [17] and the Slovenian Government Decree defining the critical infrastructures of the Republic of Slovenia and the operators of critical infrastructure of the Republic of Slovenia, healthcare institutions are considered critical infrastructure that are of vital importance to the state and whose disruption or destruction would have a serious impact on national security and the health and welfare of the population. University hospitals (University Medical Centre Ljubljana and Maribor) were granted the status of a facility of special importance for the defense of the Republic of Slovenia by a decree of the Slovenian government formulating a Defence Plan [23,24].

The COVID-19 crisis showed the importance of unhindered healthcare. Ensuring security in healthcare facilities in times of crisis is a very complex challenge that must be addressed systematically and in accordance with expertise, knowledge, and regulations. In such times of crisis, it is important to establish a crisis management system to ensure corporate security, as described above. Therefore, all activities during a healthcare crisis are managed by a crisis team appointed by the corporate security manager, who can immediately establish extraordinary management of the organization that responds immediately to the circumstances, including the establishment of basic plans for operations in emergency situations [25]. The crisis team defines all possible hazards and risks and provides practical instructions and guidelines for managing the crisis period. Another important aspect is the coordination with all internal and external actors (municipality, civil protection, ministries). Crisis management is characterized by a clear leadership hierarchy, unambiguous decision-making authority, transparent reporting responsibilities, precise timelines, and communication methods of the crisis team. This group must report comprehensively on the facts and treat all stakeholders equally when assigning tasks, informing them of the current situation, and planning activities [25].

In recent years, assaults on staff in Slovenian healthcare facilities have been reported, such as assaults on staff in one of the emergency rooms earlier this year. These incidents are rare, and more attention is paid to cyber threats, which have increased significantly in recent years, and the complexity of cyber-attacks has also increased [26]. With the advancement of digitization in healthcare, cybersecurity aspects are becoming increasingly important as sensitive and large amounts of data are processed daily [27]. Cyber security is an important component of integral corporate security that protects organizations, their technology, employees, technical resources, and data of all people in healthcare facilities from internal and external threats. Cybersecurity as a building block of corporate security includes not only the protection of confidential or personally identifiable information but also the active prevention of unauthorized or unlawful access to information and information systems and their appropriate protection from access, use, disclosure, modification, or destruction [4,28].

Cybersecurity in healthcare is in a critical state. A 2020 cybersecurity survey found that 70% of hospitals surveyed had experienced a "significant security incident" in the past 12 months, including phishing attacks and extortion software, resulting in business interruption, downtime, and financial loss [29]. Paperwork was converted to digital form and electronic database systems. Although this has proven beneficial to patients and their physicians, the industry has become very vulnerable due to greater connectivity and the resulting ease of remote access and data sharing [26]. With each advance that automation brings, the vulnerability of healthcare systems to malicious cyberattacks also increases [26].

Attacks on the healthcare sector not only threaten the security of systems and information but also directly threaten the health and safety of patients. Many healthcare organizations have various types of specialized hospital information systems, such as online appointment scheduling systems, practice management support systems, clinical decision support systems, radiology information systems, and computerized referral input systems [28,30]. In addition, there are thousands of devices that make up the Internet of Medical Things (IoMT), including all types of mobile devices and networked systems that store patient data along with medical documentation. There are numerous opportunities for data theft, so the need for a strong cybersecurity function in healthcare organizations is greater than ever [29]. The implementation of health information systems into the corporate system by healthcare providers has a positive value for managing health information but also has negative impacts, such as security and privacy risks, and therefore needs to be implemented as part of corporate security to ensure all levels of security [31].

Cybersecurity has become a critical factor in healthcare that must affect all stakeholders involved [32]. Healthcare organizations are targets of cyberattacks for three main reasons: (a) criminals on the dark web quickly and lucratively sell healthcare data for various scams, (b) the profits in the event of a healthcare system failure are very profitable, and (c) medical devices connected to the Internet are very vulnerable to unauthorized intrusion [30].

The healthcare industry may face even greater cybersecurity challenges than other industries. They need to protect the confidential financial and health data of their patients and employees, as well as their own networks, databases, and accessories [33]. New networked medical devices and accessories are entering the market every day, accounting for a significant portion of all medical devices. These networked devices are often needed to keep patients alive, and their functioning is, therefore, critical. In order to avoid legal consequences, healthcare fraud, and reputational damage due to the loss of patient data, the healthcare industry needs to address the importance of cybersecurity [34,35]. Implementing appropriate security strategies and conducting cybersecurity training as part of corporate security, where all employees learn the value of security, are therefore critical to protecting the industry. Artificial intelligence also holds tremendous potential for strengthening healthcare systems in the future and can also provide rapid insight into cyber-related threats to healthcare organizations [35].

### 1.4. The Importance of Strategic Communication of Corporate Security in Healthcare

The extent and way messages (data and information) are communicated within a public health facility and between the institution, and external stakeholders say much about attitudes within the institution and the institution’s attitude toward the environment [36]. Adequate and high-quality communication contributes to a positive climate within the institution and to efficient and successful task performance. Conversely, inadequate and poor-quality communication (formal or informal) impairs the effectiveness of operations and leads to dissatisfaction among employees and, consequently, patients. Appropriate scope and quality of the content of a message are the basis for successful communication. In terms of content, the message must be clear and as simple as possible [36].

The ability to communicate effectively is perhaps the most important of all life skills. It enables us to convey information to others and to understand what they are trying to tell us [37]. Nowadays, communication is considered an important component of healthcare. In the field of healthcare in the Western world, the acquisition of communication or interpersonal skills has become part of the training programs. Communication skills are thus embedded in the process of education and training. Communication is the foundation of healthcare, regardless of the individual’s position in the healthcare system and regardless of the healthcare discipline. This means that successful communication leads to better and higher standards of healthcare [38]. It is very important for good and effective communication, as it allows all employees to receive the same information, and only then can they work productively as a team [39].

This can also be achieved through so-called strategic communication. Strategic communication is a term that describes the communication principles, strategies, and initiatives used to promote an organization’s goals, mission, or values. It is a multidisciplinary field based on communication practice [40]. Strategic communication lies at the intersection of management (including corporate) and communication strategies and is based on the concept of purposeful communication in an institution. It can also be defined as a method of managing the non-material attributes of an organization that includes elements of communication as part of the strategy [41]. Being strategic means communicating the best message through the right channels, as measured by thoughtful organizational and communication goals [42]. Strategic communication from the perspective of ensuring corporate security thus represents a set of communication actions undertaken by an institution with the goal of creating comprehensive corporate security that ensures the security of the organization as well as the security of all employees and, in the case of healthcare institutions the safety of patients [43]. Through strategic communication, management uses them purposefully to achieve its mission.

The regulation of corporate security in facilities and the perception of employees at the primary, secondary, and tertiary levels in the Slovenian healthcare system have not been studied yet. Identifying processes and services in healthcare organizations that provide corporate security and staff familiarity with the processes is of great value to (i) obtain an assessment of the current status, (ii) identify potential caveats, and (iii) plan for necessary improvements in various areas of corporate security, with a focus on enabling physical security and cybersecurity.

## 2. Materials and Methods

Our study was designed as a descriptive cross-sectional quantitative study. According to the literature, this type of study is most used in social research and allows a group of participants to be studied only once [44]. A survey was conducted in September and October 2022 to meet the research objectives. The research design was ethical and in accordance with the Code of Ethics and Integrity for Researchers at the University of Maribor as evaluated by the Ethics Committee of The Faculty of Criminal Justice and Security, University of Maribor (protocol No. 2309–2022). The participants were anonymized in accordance with the ethical guidelines of the Faculty of Criminal Justice and Security of the University of Maribor, as well as data protection under GDPR (General Data Protection Regulation) and data protection in the Republic of Slovenia, to ensure anonymity and confidentiality of the respondents.

The survey was voluntary and anonymous. It was conducted in the online 1 ka (one-click survey) environment, and in addition, hard copies of the survey were distributed at the Security Consultation on Ensuring Corporate Security in Healthcare Facilities, held at the Faculty of Criminal Justice and Security in September 2022. The online survey and the paper version were identical in content. We emailed the online survey to all healthcare institutions in Slovenia that performed this activity at the primary, secondary, and tertiary levels and asked them to also forward the survey to colleagues who work in the field of corporate security. There are 81 of these institutions in Slovenia, from which 154 stakeholders responded. To determine the sample, we established two main criteria: (i) respondents are employed in a healthcare institution, and (ii) respondents worked in the field of corporative security in a healthcare institution in Slovenia. Thus, the criteria mainly concerned the size of the population and the confidence level to ensure that our study results were accurate, reliable, and meaningful. The results of the two forms were combined. The sampling was purposive and snowballing.

The purpose of the questionnaire was to gain insight into respondents’ perceptions of the importance of corporate security in healthcare institutions and to determine the regulation of corporate security in the institutions where respondents are employed.

The survey sought to determine what processes and services healthcare organizations currently use to provide corporate security and whether they do so themselves or through external providers. Familiarity with the legal processes of corporate security was measured, and the services they currently use to provide them were rated on a four-point Likert scale, with respondents asked to indicate how important each process is to them. The question about how corporate security is ensured was phrased in four statements, of which respondents selected the most applicable. The final section of the questionnaire contained demographic information, which is presented in this section.

Respondents’ answers were measured using a five-point Likert scale for agreement (1 = strongly disagree; 2 = strongly disagree; 3 = disagree/disagree; 4 = agree; 5 = strongly agree). To gain better insight, we also collected demographic data. 

Data were analyzed using the IBM SPSS program, version 28.0.0. In the analysis of the data obtained, the reliability of the scales was first evaluated using the internal consistency of Cronbach’s alpha, the alpha (α)-coefficient for the analysis, which represents a Type 1 error while indicating the reliability of each statement. In addition, skewness and kurtosis values were examined to determine the asymmetry of a distribution and the tail heaviness of a distribution compared to a normal distribution. Descriptive statistics methods were also used. The median, percentage (%), mean (M), and standard deviation (SD) were reported.

We also conducted a systematic literature review of available information on corporate security in healthcare facilities. The literature was systematically searched to minimize the risk of overlooking potentially relevant articles. The phases of the systematic search were conducted as described in [45,46], and our study consisted of records identified through a systematic database search, records manually isolated after database cleaning, records manually screened based on title and abstract, and full articles included in the analysis. Both corporate security and health institutions were included in the search strings, along with concept mapping and searching Thesaurus.com for synonyms to ensure that the inherent interdisciplinarity of the study was adequately captured.

## 3. Results

### 3.1. Literature Review Database Search

The systematic literature search conducted to analyze the existing body of literature identified 14 papers published till 12 April 2023. As shown in Table 1, the final search included both “corporate security” and “healthcare”, as well as “medical” or “pharma” institutions. Titles and abstracts were reviewed according to the following criteria: (i) only explicit references to health care were used, excluding any type of nonmedical use (five articles), and (ii) only articles dealing with corporate safety were selected (eight ineligible articles). The only article that was eligible was from 2014 and was published as a case report.

### 3.2. Basic Demographic Information

One hundred and fifty-four respondents participated in the study. The majority of respondents were female (57.14%, 88 respondents), while three respondents did not provide gender information.

Most of the respondents (36.36%) had a university degree (pre-Bologna process and post-secondary and higher education), while the fewest respondents had a Ph.D. (3.90%). A total of 14.94% of respondents had a secondary school degree, 22.73% of respondents had a pre-Bologna or Bologna Master’s degree, 10.39% had a post-secondary degree, and 11.69% had a Master’s degree.

The average age of respondents was 41.57 years, with an SD of 10.87 years. The minimum age was 22 years, and the oldest respondent was 67 years old at the time of the survey.

The majority of respondents in the health facility are employed or working (64.29%), while 31.82% are in a management position. A total of 51.95% of the respondents work in the field of nursing, while 12.99% work in human medicine. The rest (12.34%) hold a managerial position, are employed in the Ministry of Interior, or work in private/technical security or IT security services. Most respondents are employed in tertiary health care institutions (41.56%), 26.62% in secondary health care, and 16.88% in the primary level of health care. The remaining respondents (14.94%) do not work at any level of healthcare but are primarily employed in leadership, management, or operational roles in corporate security.

According to the analysis results, both skewness and kurtosis were within the range (−2,2), proving that the data had a normal distribution and were suitable for parametric testing. In addition, the reliability of the data was measured by Cronbach’s coefficient α, which indicated high reliability of the data with a value of 0.834 (Table 2).

### 3.3. Procedures to Ensure Corporate Security in Healthcare Institutions

Legal assurance of uninterrupted operation, legal assurance of security know-how, legal assurance of IT security, legal protection of rights, and legal assurance of private security and a safe workplace. Accordingly, the survey highlighted the processes that build comprehensive corporate security and the procedures established. Respondents rated each process based on how important it was to their organization.

A total of 44.16% (68 respondents) indicated that legal support for processes related to the implementation of corporate security at their institutions was partially important, but indicated that additions were needed in this area. On the other hand, a few respondents (11.69%) rated processes related to corporate security implementation at their institution as not important (Table 3). Most of the respondents rated this process as partially important (mode = 2), while the opinion of the respondents in relation to mean (x = 2.49), assuming that it is normally distributed in the sample, is evaluated in intervals of 1.58 and 3.32 (SD = 0.902) (Table 4). 

Similar results were obtained for the area of licensing, where 30.52% of the respondents considered licensing in their institutions to be partially important, while 27.92% thought that it was sufficiently well-regulated and that only minor adjustments were needed. The mean of this statement was 2.53 (x = 2.53) with a standard deviation of 1.049 (SD = 1.049).

When asked about physical and technical security, most respondents rated this area as partially or sufficiently important for their healthcare institution (from 33% to 27%). Only a small percentage—5.84% for technical security and 14.49% for physical security rated this area as not important for their organization. 

In addition, 43.51% (67 respondents) rated security consulting as partially important to their facility, and relative to the mean (x = 2.40), respondents’ opinions ranged from 1.44 to 3.36 (SD = 0.959).

Most respondents rated that insurance against damage was partially important for their facility, with the need to add it (mode = 2).

Finally, regarding ensuring IT security, 37.01% of respondents considered that ensuring the security of IT was sufficiently important for the healthcare institutions where they work (mode = 3) (Table 3 and Table 4).

The mean values for the processes to ensure corporate security were in an interval of 2.49 to 2.84, while the mode was higher only for ensuring the security of IT with a value of 3. The median value obtained was in an interval of 2 to 3, which means that half of the respondents rated the importance of the defined processes as 2 or 3 less, and the other half rated them as 2 or 3 more (Table 4).

### 3.4. Methods Used to Ensure Corporate Security in Healthcare Facilities

Further on, participants were asked about the method to ensure corporate security in healthcare facilities; respondents chose from four possible methods, as shown in Table 5. 

The results showed that 52.60% of the respondents indicated that in-house corporate security was the most used, some of which are provided by external providers. In 25.32% of the responses (39 respondents), it was indicated that most of the corporate security in the healthcare institution where they are employed is provided by external providers, and a smaller part is covered by internal security. Only 8.44% (13 respondents) indicated that corporate security is provided solely by an external provider, and only 13.64% (21 respondents) indicated that it is provided entirely by their company’s own corporate security facility (Table 5).

If all respondents in the sample gave the same assessment of the method used to ensure corporate security, it would tend to be that they ensure it mostly themselves and partly through external providers (x = 2.29), while half of the respondents gave the assessment that it is either ensured themselves or mostly ensured themselves and partly through external providers, and the other half gave the assessment that it is mostly ensured by external providers and only partly ensured themselves or entirely by external providers (median=2). In terms of the median, therefore, the middle 68% of respondents chose an answer in the interval between 1.49 and 3.10. The (min–max) IQR (Interquartile Range) was calculated and is presented in the Table 6. Most respondents chose the answer that they ensure corporate security themselves and partly through external providers (mode = 2) (Table 6).

## 4. Discussion 

The corporate security strategy is increasingly becoming an approach that institutions can use to identify and manage or at least reduce risk [39]. Although it has been around for a long time, corporate security has become more important in modern times since the 2001 terrorist attack in New York [47], and its importance is also increasing with the development of technology itself and the digitalization processes in the public and private sectors. As new technologies emerge, so do new types of threats that can jeopardize the unimpeded operation of institutions [5]. Organizations, including healthcare institutions, are becoming more dependent on it, and it is important that they develop the kind of corporate security that prevents accidental and malicious events that threaten the accessibility, authenticity, and confidentiality of stored data [48]. It has been shown that organizations with a well-functioning security system operate more efficiently and are able to overcome business and security challenges [12,47].

Reviewing electronic databases, we did not find any research of this type on this topic internationally or in Slovenia, which makes it even more important to point out the importance of this topic and the benefits of implementing corporate security in healthcare institutions. The literature search revealed only one case report evaluating corporate security in one institution [49]. Corporate security reduces security risks and prevents losses in organizations. At the same time, it is important that all employees are committed to the highest possible safety and security culture, which can be ensured through proper strategic communication. Implementing corporate security processes in healthcare institutions facilitates their uninterrupted operation and, most importantly, enables the organization to be truly secure and able to respond to crises or unforeseen situations. Thus, appropriate and comprehensive corporate security in healthcare serves to comprehensively protect organizations by ensuring lawful and unhindered operations, protecting against risks and threats, ensuring information security, and ensuring physical and technical security.

Our survey, which included 154 stakeholders from all healthcare sectors, revealed that employees are aware of the importance of corporate security and that ensuring security in healthcare institutions is not yet complete or comprehensive. Respondents ranked certain business processes as important to corporate security but have not yet implemented them in their organizations, or they are partially implemented by external providers. The research conducted showed that corporate security is present in Slovenian healthcare facilities but that additional efforts are needed to ensure it. This is also due to the current challenges in the Slovenian healthcare system, which are related to both the measures taken after the COVID-19 epidemic and the shortage of healthcare personnel. The research has shown that the legal processes of corporate security in healthcare organizations are mostly moderately ensured and that these processes are partially relevant for the institutions, but further additions are needed. The most important factor in ensuring that these processes are in place and relevant is to remove administrative barriers. This would allow the processes to be truly aligned with ensuring security and set up quickly and appropriately depending on the current risks. Individual corporate security processes are currently provided primarily by internal providers, but it would make sense to outsource certain processes to external providers or independent experts. In this way, healthcare institutions could transfer some of the risks to others and resolve certain risks in a systematic and truly competent manner. It would also make sense for the internal providers to be monitored by independent commissions.

We see the importance of linking the areas in multidisciplinary collaboration in corporate security in healthcare organizations, reinforced by ensuring safety as an additional discipline, which, according to Maslow, is one of the basic human needs and allows employees to function normally in crisis situations and patients to recover normally.

Even though the survey showed that healthcare institutions attach the greatest importance to information security compared to other legal procedures of corporate security and are aware of its importance, the actual level is still too low, given the current cyber threats. Further improvements are needed, especially training and education of the entire workforce, as this is the only way to ensure comprehensive and systemic corporate security. Despite globalization, we must not forget that people still play the most important role in ensuring security. Comprehensive corporate security, therefore, depends on human action. Therefore, it is important that the established legal processes are appropriately and strategically communicated to all stakeholders.

Zafair et al. [49] reviewed the literature on effective security management programs (STM) in organizations and conducted a survey on the perceived effectiveness of the SRM program in a large healthcare institution. Results indicated that employees are aware of SRM policies, but the program may not be as effective as it should be. The use of sophisticated technologies in healthcare facilities in Slovenia also increases the risks associated with their information. Corporate security in healthcare facilities also uses the STM as the basis for creating the security plan, which is the responsibility of the corporate security manager and is not available to all security staff, so we were unable to compare the results of the two studies.

A successful security strategy requires employee training because only a professionally empowered employee can meet security challenges. The security manager must raise employee motivation and awareness that security in the company applies to everyone and that a successful security climate requires that every employee do whatever is necessary to contribute to the security of the organization. Security companies are committed to private sector security, but this cannot be performed without the cooperation of law enforcement. When security companies uncover criminal activity, they must involve the police, who are responsible for such security risks. For a successful security policy, security companies could also cooperate with the police when it comes to advice and experience sharing because the police are considered the greatest expert in the field of security; they could share their knowledge and tactics with security companies, which would incorporate this advice into their corporate security [19].

It is critical for healthcare institutions to ensure lawful and unhindered operations, and corporate security mechanisms ensure this by adequately addressing security issues and corporate actions. Equally important to ensuring unimpeded operations is the legal regulation of trade secret protection and anti-corruption and employee integrity procedures. Implementing and securing expertise focuses on investigating and detecting illegal or deviant behavior in healthcare facilities, which can be supported by building OSINT (Open Source Intelligence). Given the evolution of technologies and associated threats, establishing cybersecurity is extremely important for any organization, and in healthcare institutions, the focus is on ensuring the security of all data (from employees and especially patients). This can be ensured by adopting appropriate bylaws and regular monitoring of cybersecurity mechanisms [11]. Legal protection of material rights and intellectual property rights in healthcare includes securing and protecting certain patents and, in particular, medical devices, where it is important that they function smoothly in all situations and are not exposed to any threat, physical or virtual [1].

Our study, which presents the current state of corporate security management in healthcare institutions, has provided answers to important questions to determine the current state. Although this study provided rich data from both quantitative and qualitative research, it would be possible to include more questions and more different types of employees in the study. Since the results showed that people are not well acquainted with the entire concept, we would like to continue to actively work to familiarize people with the concept and increase people’s awareness. In addition, it would be interesting to survey a wide range of employees, compare the status with the changes made based on this study, and additionally measure the level of safety culture in the healthcare organization. Safety culture is sometimes referred to as safety and security culture. Safety culture refers to the values, attitudes, beliefs, and behaviors within an organization that prioritize and promote the safety of its employees, customers, and stakeholders. It is a collective mindset that emphasizes the importance of identifying and mitigating potential hazards, risks, and threats to prevent harm. A strong safety and security culture requires leadership commitment, employee engagement, and continuous improvement. It requires that all members of an organization, from top management to frontline employees, actively participate in identifying potential risks and threats and taking appropriate measures to mitigate them. This can include developing and enforcing safety policies and procedures, providing regular training and education on safety and security best practices, conducting regular audits and inspections, and fostering a culture of open communication and sharing. In healthcare institutions, a strong security culture involves creating an environment where all employees take responsibility for protecting sensitive patient data. This can be achieved through a combination of training, policies, and technology.

Training is an essential part of building a security culture in healthcare institutions, which is also part of the security plan. All employees should receive regular training on how to recognize and respond to security threats, such as phishing attacks and ransomware. They should also be educated on the importance of protecting patient data and the consequences of not doing so. Technologies such as firewalls, antivirus software, and encryption can play an important role in promoting a culture of security in healthcare institutions. These technologies can help to protect sensitive patient information and prevent unauthorized access.

By prioritizing safety and security culture, organizations can create a safer work environment, reduce the risk of accidents, incidents, and security breaches, and ultimately protect their employees, customers, and reputation.

During epidemics, corporate security in healthcare institutions focused on monitoring compliance with security measures put in place by the government to curb the spread of infectious diseases. Monitoring of compliance with security measures was conducted by security institutions. The police primarily monitored compliance with security measures in public, while the security services monitored compliance with security measures within the various institutions (The new normal 2.0: Private security and COVID-19 in Europe, 2020). From a security perspective, it is also important for corporate security to participate in the One Health concept, as it is the only way to have a security plan and security insights that institutions can consider during epidemics to help contain the spread of infectious diseases. 

Even as the coronavirus spread during the epidemic, successful and effective strategic communication was required between organizations fighting the spread of the new coronavirus. To effectively protect the community, law enforcement agencies needed to develop effective and meaningful methods of communicating with local hospitals, health departments, security services, and other organizations to pool knowledge and resources. Through effective joint communication, these organizations could also raise public awareness of the protocols of social distancing, isolation, quarantine, and any other measures relevant during the epidemic [48]. Strategic communication and corporate security are both important aspects of healthcare facilities, even on a day-to-day basis. Effective communication is necessary to build trust and credibility with patients, staff, stakeholders, and the broader public. At the same time, healthcare facilities must ensure the security of their operations, data, and physical assets. 

To achieve these goals, healthcare institutions can take several actions, such as (i) developing a communications strategy; healthcare institutions should develop a communications strategy that outlines their goals, target audiences, key messages, and communication channels. This strategy should be aligned with the overall mission and values of the institution. (ii) Staff training: healthcare institutions should regularly train their staff on effective communication techniques, privacy and security policies, and emergency protocols. (iii) Implement security measures: Healthcare institutions should implement robust security measures to protect their data, systems, and physical assets. These measures could include firewalls, encryption, access controls, and monitoring systems. (iv) Conduct regular risk assessments: Healthcare institutions should regularly assess the risks to their operations, including risks from cyberattacks, physical breaches, and other security threats. This will help them identify vulnerabilities and take corrective action before an incident occurs. (v) Stakeholder engagement: Healthcare institutions should engage with their stakeholders, including patients, employees, regulators, and the broader public, to build trust and credibility. This could include regular communication, feedback mechanisms, and public engagement programs. By implementing these measures, healthcare institutions can ensure they have effective communication strategies in place and security measures in place to protect their operations and stakeholders.

We can conclude that ensuring adequate corporate security in healthcare is a complex process that requires extensive knowledge and processes that are implemented in dependence on all other key corporate functions. Therefore, good corporate security is a foundation for the unhindered operation and business of healthcare institutions [19]. It is one of the fundamental functions for the operation of healthcare institutions, and for its effective functioning, its implementation in close connection with all key functions in the company is essential. The basic objective of the corporate security system is to ensure the internal security of the company. This is achieved through a series of measures at the legal, organizational, functional, technical, and personnel, which must be aimed at compliance with laws and regulations and ensuring the safety of persons and property in healthcare institutions.

## 5. Conclusions

Healthcare institutions have special security needs that must be met. Therefore, they should employ physical and electronic security measures, as well as strategic communications, to ensure that their enterprise-wide security policies and procedures are properly implemented. They should also ensure that they comply with applicable laws and regulations to protect the interests of their patients and employees. It is important to be aware that corporate security in healthcare institutions is essential for the safety of patients, staff, and medical records. Healthcare facilities must implement a comprehensive security strategy that protects their physical and digital premises as well as the sensitive data in their systems.

## Figures and Tables

**Table 1:** Literature review database search.

Search Query	Corporate Security AND (Healthcare OR Medical or Pharma) Institutions
Databases:	Scopus
Keywords in:	Title, abstract, keywords
Year restriction	None
Language restriction	English
Subject areas	Medicine; Social sciences, Management; Business; Security

**Table 2:** Reliability coefficient of analyzed data.

Reliability Statistics	N of Items	Cronbach α
	8	0.834

**Table 3:** Assessing the importance of corporate security assurance processes in healthcare organizations.

Processes of Ensuring Corporate Security in Healthcare (N = 154)	Process Is Not Significant for Their Institution	Process Is Partly Significant for the Institution, but Needs Supplementing	Process Is Partly Significant for the Institution, but Needs Minor Supplementation	Process Is Very Significant for the Institution, and This System Just Needs to Be Maintained
Legal support of all corporate processes	11.69%	44.16%	27.92%	16.23%
Properly arranged licensing	19.48%	30.52%	27.27%	22.73%
Physical security	14.94%	33.77%	27.92%	23.38%
Technical security	5.84%	33.12%	31.82%	29.22%
Security consulting	16.88%	43.51%	22.73%	16.88%
Insurance against damage events	8.44%	42.86%	28.57%	20.13%
Ensuring IT security	5.19%	30.52%	37.01%	27.27%

**Table 4:** Descriptive statistics for processes that are significant for ensuring corporate security.

Ensuring Corporate Security in Healthcare (N = 154)	Mean	Median	Mode	SD
Legal support of all corporate processes	2.49	2	2	0.902
Properly arranged licensing	2.53	2.5	2	1.049
Physical security	2.6	3	2	1.007
Technical security	2.87	3	2	0.916
Security consulting	2.4	2	2	0.959
Insurance against damage events	2.6	2	2	0.903
Ensuring IT security	2.86	3	3	0.879

**Table 5:** Methods used to ensure corporate security.

How Healthcare Organizations Ensure Corporate Security (N = 154)	%
They ensure it entirely themselves	13.64%
For the most part, they ensure it themselves, and partly external providers	52.60%
For the most part, it is ensured by external providers and partly by themselves	25.32%
It is ensured entirely by external providers	8.44%

**Table 6:** Descriptive statistics for the methods of ensuring corporate security.

Ensuring Corporate Security in Healthcare (N = 154)	Median	Mode	Min/Max	IQR
Method of ensuring corporate security	2	2	1/4	25% = 2 50% = 2 75% = 3

## Data Availability

The data presented in this study are available on request from the corresponding author. The data are not publicly available due to GDPR and ethical approval of Faculty of Criminal Justice and Security.
